# Acetabular Component Positioning Using the Transverse Acetabular Ligament as an Anatomical Landmark in Primary Total Hip Arthroplasty: A Prospective Study

**DOI:** 10.7759/cureus.81940

**Published:** 2025-04-09

**Authors:** Akshay R, Supreeth D R., Aravind J Devendrappa, Ambareesh Parameshwar, Aishwarya Megnath, Mohammed Shahid

**Affiliations:** 1 Orthopedic Surgery, Chamarajanagar Institute of Medical Sciences, Yadapura, IND; 2 Orthopedic Surgery, Vydehi Institute of Medical Sciences and Research Centre, Bengaluru, IND

**Keywords:** acetabular cup positioning, anteversion, inclination, postoperative ct scan, total hip arthroplasty, transverse acetabular ligament

## Abstract

Introduction

Total hip arthroplasty (THA) is a well-established procedure aimed at relieving pain and enhancing mobility in patients suffering from various hip pathologies, such as avascular necrosis (AVN), post-traumatic arthritis, ankylosing spondylitis (AS), and juvenile rheumatoid arthritis (RA). Precise placement of the acetabular cup is critical to reduce joint wear, dislocation, and component loosening, ultimately enhancing patient outcomes. The optimal positioning of the acetabular cup involves anteversion between 5° and 25° and inclination between 30° and 50°. The transverse acetabular ligament (TAL) has been proposed as a reliable anatomical landmark to guide cup placement in primary THA. This study evaluates the accuracy of acetabular component positioning using TAL as a reference by analyzing post-operative inclination and anteversion angles via CT scans.

Methodology

A prospective study was conducted at the Department of Orthopaedics, Vydehi Institute of Medical Sciences and Research Centre, Bengaluru, India, involving 27 patients (22 males, 5 females) aged 18-80 years undergoing primary THA with TAL as the guiding landmark. Patients with revision hip arthroplasty, prior acetabular fractures, or surgeries around the hip were excluded. During surgery, the acetabular cup was aligned parallel to the TAL using the posterolateral approach. Post-operative CT scans were performed to assess the inclination and anteversion angles. Descriptive statistical analysis was conducted using IBM SPSS Statistics for Windows, Version 23 (Released 2016; IBM Corp., Armonk, New York, United States) with significance set at p < 0.05.

Results

The mean inclination angle measured post-operatively was 43.4° ± 4.5°, while the mean anteversion angle was 20.8° ± 4.4°. These values were found to be statistically significant at a 1% level of significance (p < 0.001). The majority of patients (55.7%) underwent THA due to AVN, followed by post-traumatic arthritis (18.6%), AS (11.3%), RA (11.3%), and neck of femur (NOF) fracture (3.1%). The results demonstrate that using TAL as a landmark provides a reliable technique for achieving optimal acetabular cup orientation.

Conclusion

The use of the TAL as an anatomical guide during THA effectively positions the acetabular component within the recommended safe zones of inclination and anteversion. This technique offers a reliable, reproducible method for improving surgical outcomes and minimizing post-operative complications. Further research with larger cohorts and multi-center trials is recommended to validate these findings.

## Introduction

Sir John Charnley, a British Orthopaedic surgeon, is credited with pioneering the fundamental principles of the artificial hip and is often referred to as the father of total hip arthroplasty (THA) [1]. THA stands as a highly effective orthopedic intervention widely practiced today. This procedure proves invaluable for patients grappling with hip discomfort, offering pain relief, functional restoration, and enhancement of quality of life. Numerous underlying causes contribute to hip arthritis, encompassing conditions such as avascular necrosis (AVN) of the femoral head, post-traumatic arthritis, ankylosing spondylitis (AS), and juvenile rheumatoid arthritis (RA) [1]. The demographic of patients undergoing THA is evolving over time. Previously dominated by elderly individuals with limited activity, it now includes younger patients seeking to regain full function similar to healthy adults. Socio-cultural factors necessitate activities like squatting and sitting cross-legged, which are crucial for these patients. Surgeons face a unique challenge in meeting patient expectations for excellent outcomes while employing evidence-based and cost-effective implants [2].

The transverse acetabular ligament (TAL) is a part of the acetabular labrum, which bridges the acetabular notch. TAL has been used as an anatomical guide for aligning cup anteversion during THA. Incorrect positioning of the acetabular cup can result in various short-term complications, such as post-operative hip joint dislocation, component impingement, and limb length discrepancy, as well as long-term issues like increased wear and tear, osteolysis, and loosening [3]. Studies indicate a considerable decrease in dislocation rates when the acetabular cup's anteversion falls within 5-25 degrees and inclination ranges between 30 and 50 degrees. This range, coined as the "safe zone" by Lewinnek, delineates the optimal placement of the acetabular cup based on anatomical considerations and biomechanical principles [4].

The orientation of the acetabular component following THA is characterized by two angles: the inclination angle and the anteversion angle. These were typically measured using radiographs. However, recent studies indicate that CT-based measurements offer greater accuracy. This superiority is attributed to variations in pelvic tilt, pelvic rotation, and component inclination [5]. This study was conducted to understand the accuracy of acetabular cup positioning when TAL is used as a landmark by measuring the inclination and anteversion angles of the cup with the help of a post-operative CT scan in THA.

## Materials and methods

This prospective study was conducted in the Department of Orthopaedics at Vydehi Institute of Medical Sciences and Research Centre, Bengaluru, India, from 01 April 2021 to 30 November 2022, following its approval by the Vydehi Institutional Ethics Committee with the approval number VIEC/PG/APP/029/2020-21. A total of 27 patients who required THA and met the inclusion criteria were recruited for the study. The inclusion criteria encompassed patients between 18 and 80 years of age, of both sexes, presenting with conditions such as RA, AS, post-traumatic arthritis, osteoarthritis, AVN, and neck of femur (NOF) fracture requiring THA. Patients undergoing revision hip arthroplasty, those who did not consent to participate, and patients with a history of previous acetabular fractures, pelvic fractures, or previous surgeries around the hip were excluded from the study.

Preoperative evaluation included a detailed clinical assessment, necessary preoperative investigations, and pre-anesthetic evaluation as required. The surgical procedure was performed under combined spinal and epidural anesthesia, with patients positioned in the straight lateral decubitus position. A slightly curved incision was made proximally at a point level with the anterior superior iliac spine, extending along a line parallel to the posterior edge of the greater trochanter. The posterolateral approach was utilized to expose the hip joint by dissecting and retracting subcutaneous tissue, fascia, muscles, and the posterior capsule. The hip was dislocated using flexion, adduction, and internal rotation maneuvers. Femoral neck osteotomy was performed, and the femoral head was removed along with the attached soft tissues. Acetabular preparation was carried out using motorized reamers with gradually increasing sizes by 1 or 2 mm, ensuring reaming was parallel to the TAL. The acetabular cup was positioned parallel to the TAL intraoperatively in all cases. Following adequate preparation, trial acetabular and femoral components were inserted, and intraoperative stability, along with the range of motion, was assessed. After confirming appropriate alignment, the final implantation of components was performed, and the surgical wound was closed in layers following thorough irrigation.

Post-operatively, all patients underwent a CT scan of the pelvis in the supine position to measure the inclination and anteversion angles of the acetabular cup. The inclination angle was measured on coronal CT sections by drawing a horizontal line connecting the inferior pubic rami and a line passing through the acetabular cup. The angle between these lines was recorded as the inclination angle. Anteversion was measured on transverse CT sections at the acetabular level by drawing a line connecting the anterior and posterior edges of the cup, with the angle formed by this line and the horizontal pelvic axis recorded as the anteversion angle [3]. The sample size is calculated by using the following formula,

\[ N = \frac{Z^2_{1-\alpha/2} \cdot \sigma^2}{d^2} \]

Where $Z_{1 - \alpha / 2}$ = 1.96 at 95% confidence level, (d/σ)^2^ = 0.4 effect size. The minimum sample size obtained was 24. With a 10% attrition rate, the sample size obtained is 27. Statistical analysis was performed using IBM SPSS Statistics for Windows, Version 23 (Released 2016; IBM Corp., Armonk, New York, United States). Descriptive analysis of explanatory and outcome parameters was carried out using mean and standard deviation for quantitative variables, while frequencies and proportions were used for categorical variables. A p-value of less than 0.05 was considered statistically significant.

## Results

The study involved 27 patients who met the inclusion criteria. The gender distribution of data was 22 male patients (81.6%) and five female patients (18.4%) (Table 1).

**Table 1:** Gender-wise distribution of study participants

Gender	Number	Percentage (%)
Male	22	81.6
Female	5	18.4
Total	27	100

The mean age of male patients was 42.6 ± 11.1 years. The mean age of females was 33.4 ± 15.6. Total hip replacement was done for varied causes, including three cases of AS (11.3%), 15 cases of AVN (55.7%), five cases of post-traumatic arthritis (18.6%), three cases of RA (11.3%) and one case of NOF fracture (3.1%) (Table 2).

**Table 2:** Number of patients based on the preoperative diagnosis

Diagnosis	Number	Percentage (%)
Ankylosing spondylitis	3	11.3
Avascular necrosis	15	55.7
Post-traumatic arthritis	3	18.6
Rheumatoid arthritis	3	11.3
Neck of femur fracture	1	3.1
Total	27	100

**Figure 1:**
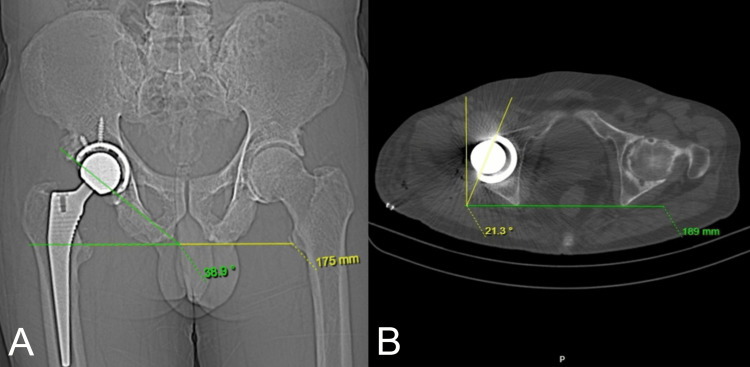
CT scan used to measure acetabular component positioning (A) Coronal section showing acetabular component inclination (B) Axial section showing acetabular component anteversion

The patients were assessed using the CT scan post-operatively to measure the angle of inclination and angle of anteversion (Figure 1).

**Figure 2:**
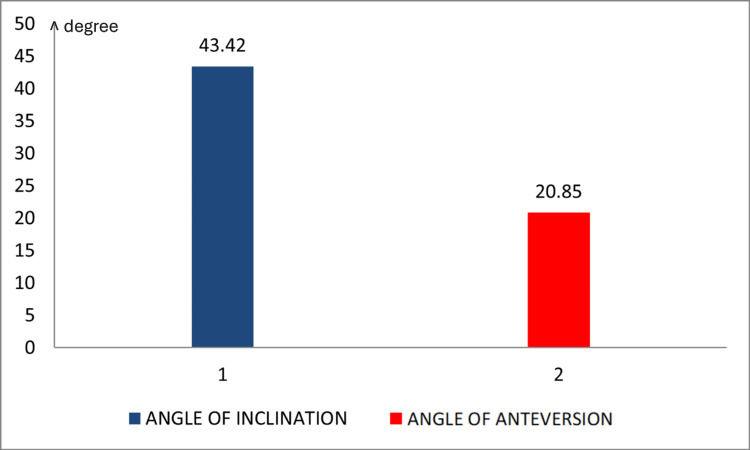
Post-operative mean inclination and anteversion

In the TAL group, the acetabular cup was positioned parallel to TAL intraoperatively in all the patients. The post-operative mean inclination measured by CT scan was 43.4 ± 4.5 degrees, and the mean anteversion was 20.8 ± 4.4 degrees. These values were found to be statistically significant at the 1% level, with p < 0.001 (Figure 2).

## Discussion

The anatomical, operative and radiographic orientations are the three most important aspects which help in predicting/providing the orientation in terms of inclination as well as the anteversion. It has been seen that almost 80% of the dislocations after primary hip replacement occur within the first two months after surgery. A high inclination angle has been associated with an increased rate of dislocation [6]. Sotereanos et al. [7] reported a reduction in dislocation rates to less than 1% when anatomical landmarks were used to accurately position the acetabular component. Ensuring the correct version of the cup is crucial, as it directly impacts its placement in other planes.

Our research included 27 patients, with 22 (81.6%) being males and five (18.4%) being females. Our findings revealed a clear predominance of males. However, contrasting results were observed in certain studies where the majority of subjects were females. Inoue et al. [8] had 19 females (65.5%) and 10 males (34.5%) in their study. Nishino et al. [9] had 45 females (75%) and 15 males (25%) in their study. Despite the literature indicating a higher incidence of total hip replacement in females due to factors such as reduced sunlight exposure, lower vitamin levels, and increased susceptibility to co-morbidities like diabetes stemming from decreased outdoor physical activity, our study suggested a higher prevalence in males. This could be attributed to factors such as accidents, wear and tear, and improper alignment during workout sessions or outdoor activities. The mean age of male patients was 42.6 ± 11.1 years. The mean age of females was 33.4 ± 15.6. While the demand for hip replacement surgery is typically high among the elderly due to joint wear and tear, there's been a notable rise in younger individuals requiring this procedure. This trend can be attributed to factors such as trauma, shifts in lifestyle choices, increased participation in strenuous activities like sports and workouts, increased awareness, expectations and availability of improved healthcare facilities.

A diagnostic analysis of our study revealed that AVN of the hip was observed in 52.94% of cases, followed by post-traumatic arthritis in 17.65%, AS and RA each in 11.76%, and NOF fracture in 5.88%. Agarwal et al. [3] found in their study that the most common causes were AVN of the hip (40%), NOF fracture (34.3%), post-septic sequelae (20%), and primary osteoarthritis (5.7%). However, findings from other studies indicated that wear and tear, alongside uniform structural loss, were contributing factors leading to functional impairment in affected individuals. Nishino et al. [9] recorded a preoperative diagnosis of osteoarthritis in 47 hips and osteonecrosis of the femoral head in 19 hips. Archbold et al. [10] categorized four types of TAL encountered during THA. The most prevalent type was those directly visible upon exposure of the acetabulum, followed by those covered by soft tissue that could be readily removed through blunt dissection. The third type involved TAL concealed beneath an osteophyte, and the least common was when no TAL was present. It was found that there is only a moderate interobserver agreement and intra-observer reliability in the alignment of the acetabular component using the TAL and the posterior labrum [10]. Thus, it can be said that even though TAL is a patient-specific landmark, its use to place the cup can have variations between the surgeons.

In our study, among patients in the TAL group, the acetabulum cup was consistently positioned parallel to the TAL intraoperatively. Post-operatively, the mean inclination measured by CT scan was 43.4 ± 4.5, and the mean anteversion was 20.8 ± 4.4. This difference was statistically significant at a 1% level of significance. Agarwal et al. [3] reported a mean inclination of 44.8 ± 4.9 and mean anteversion of 23.8 ± 4.9 in their study using TAL as a landmark, suggesting its usefulness as a patient-specific guide for acetabular cup placement. Idrissi et al. [11] recorded a mean inclination angle of 39.85 ± 5.0 and a mean anteversion angle of 16.9 ± 5.0, concluding that TAL can aid in positioning the acetabular cup in total hip replacement. Pazhani and Sathish [12] found a mean inclination of 41.4 ± 7.15 in their study and suggested TAL as a reliable intraoperative landmark for maintaining cup inclination when positioned within 6 mm from the TAL. Inoue et al. [8] used TAL as a landmark for acetabular anteversion and recorded a mean inclination of 41.5 ± 4.60 and mean anteversion of 26.5 ± 8.9. For accurately measuring post-operative inclination and anteversion, CT scans are preferred due to their ability to account for variations in pelvic tilt, pelvic rotation, and component inclination. It has been suggested that CT remains the more precise modality for this purpose [5,13]. In a study by Yoon et al. [14], the results showed 8.8% of hips post-THA were outside of Lewinnek's safe zone, and there was a close relationship between the acetabular anteversion and TAL anteversion. In a similar study by Meermans et al. [15], the mean angle of anteversion for women in the TAL group (18.2°; 9° to 25°) was significantly larger than in men (15.1°; 5° to 23°) (p = 0.03) with a significantly lower variation in anteversion when using the TAL. The findings of our study also align with the systematic review by Ning et al. [16], which concluded that the TAL serves as a reliable intraoperative landmark for acetabular component positioning within Lewinnek’s safe zone. Our results (mean inclination: 43.4° ± 4.5°, anteversion: 20.8° ± 4.4°) corroborate their observation that TAL-guided placement achieves angles consistent with reduced dislocation risks. Though our study had no cases where the acetabular anatomy was deranged like in dysplasia, according to the study by Epstein et al. [17], the TAL may not be a reliable landmark for acetabular component positioning in dysplastic hips. In their study, the TAL was not consistently identifiable in all patients, particularly in those with abnormal acetabular anatomy, such as dysplasia. Surgeons failed to locate the TAL intraoperatively in 37% of cases (14/38 hips), many of which involved dysplastic or complex anatomies. Even when identified, the TAL-guided cup placement in dysplastic hips did not reliably achieve angles within Lewinnek’s safe zone.

This study, however, has some limitations that should be acknowledged. Firstly, the study involved a relatively small sample size of 27 patients, which may limit the generalizability of the findings. A larger cohort would provide more robust data and improve the statistical power of the results. Secondly, the study was conducted at a single center, which may introduce bias and limit the applicability of the findings to broader populations. Multi-center studies are required to validate the use of the TAL as a reliable landmark for acetabular cup placement in diverse patient groups. Additionally, the study did not account for potential intra- and interobserver variability in the assessment of TAL and measurement of post-operative angles, which could affect the reproducibility of the results. Furthermore, the lack of long-term follow-up precludes the assessment of potential complications or component failures that may occur over time. The focus was primarily on immediate post-operative outcomes, and the longevity of the prosthesis aligned using TAL remains to be evaluated. Finally, factors such as differences in patient anatomy, variations in surgical technique, and differences in implant design were not thoroughly considered, which could influence the positioning of the acetabular component. Future studies should aim to address these limitations by including a larger sample size, multiple study centers, and long-term follow-up assessments.

## Conclusions

This study demonstrates that the TAL serves as an effective and reliable anatomical landmark for acetabular cup placement in primary THA. The mean inclination and anteversion angles achieved, 43.4 ± 4.5 and 20.8 ± 4.4, respectively, fall well within the safe zones, suggesting that TAL can be used to achieve optimal cup orientation. Despite the encouraging results, the study’s limitations, particularly its small sample size and single-center design, underscore the need for further research. Larger, multicenter studies with long-term follow-up are essential to validate these findings and assess the durability and clinical relevance of using TAL for acetabular component positioning. Additionally, the standardization of techniques to reduce observer-related variability should be explored. In conclusion, while TAL appears to be a promising guide for acetabular cup placement in primary THA, further research is necessary to confirm its reliability and improve its application in clinical practice.
